# A Displacement Sensing Method Based on Permanent Magnet and Magnetic Flux Measurement

**DOI:** 10.3390/s22124326

**Published:** 2022-06-07

**Authors:** Jikai Zhang, Yicheng Shi, Yuewen Huang, Cheng Liang, Yantong Dong, Yihua Kang, Bo Feng

**Affiliations:** 1 National Center for Dam Safety Engineering Technology Research, Changjiang River Scientific Research Institute, Wuhan 430010, China; jikaizhang@hust.edu.cn (J.Z.); huangyw@mail.crsri.cn (Y.H.); 2 Three Gorges Construction Engineering Corporation, Beijing 101100, China; shi_yicheng@ctg.com.cn; 3 Institute of Science and Technology, China Three Gorges Corporation, Beijing 100036, China; lcmeijin@163.com (C.L.); dong_yantong@ctg.com.cn (Y.D.); 4 School of Mechanical Science and Engineering, Huazhong University of Science and Technology, Wuhan 430074, China; yihuakang@hust.edu.cn

**Keywords:** displacement sensor, magnetic flux, permanent magnet

## Abstract

This paper proposes a displacement sensing method based on magnetic flux measurement. A bridge-structured magnetic circuit, formed by permanent magnets and two ferromagnetic cores, is designed and discussed. The analyses of the equivalent magnetic circuit and three-dimensional finite element simulations showed that the magnetic flux density changes linearly with the reciprocal of the sum of a constant and the displacement. A prototype sensor of the bridge structure is developed that consists of four permanent magnets as excitation, a Hall sensor as reception, and two ferromagnetic cores as the connection. Experiments have validated the feasibility of this method. The measured results show a good linearity between the sensor’s output and the reciprocal of the sum of a constant and the displacement, with a correlation coefficient greater than 0.9995 across different measurement ranges. Additionally, the measured results significantly indicate that the proposed sensor is compatible with different ferromagnetic materials with a worst-case error of less than 5%. The proposed sensor has the advantages of low cost and good linearity; however, the test object is limited to ferromagnetic materials.

## 1. Introduction

Displacement measurement techniques have been widely applied in metrology and many other industrial fields. The need for displacement measurements in various applications has been increasing over the past few decades [1]. Various displacement sensors, such as capacitive sensors, eddy current sensors (ECSs) and optical sensors have been developed. Capacitive sensors are typically divided into two categories: variable-area and variable-gap types. The former has a larger measurement range and lower sensitivity than the latter [2]. The development of long-range sensors with high sensitivity is the main objective of the design of capacitive sensors. High-performance capacitive sensors with a measurement range of centimeters and resolution of nanometers have been reported [3,4,5]. However, the application of capacitive sensors is limited by their vulnerability to dust and water in the environment [6]. ECSs are based on electromagnetic induction and have been widely applied in nondestructive testing [7,8,9,10]. Conventionally, an ECS is composed of an excitation and a reception component. High-frequency excitation induces eddy currents on or near the surface of the metal, and induces a strong secondary magnetic field [11,12]. Variations in displacement change the intensity of eddy currents and the corresponding magnetic field. ECSs are inexpensive, robust and immune to harsh environments [1,13,14]. However, to ensure high sensitivity, high excitation frequency should be used. The use of high frequency leads to the degradation of the resistance to electromagnetic interferences [15]. Compared with these sensors, optical displacement sensors have the best electromagnetic compatibility in complicated electromagnetic environments [16,17,18]. Owing to their high precision and large measurement ranges, optical sensors, especially optical fiber displacement sensors, have attracted much attention in many fields. However, their high prices and complex structures limit the applications [17,19,20].

Magnetic sensors have been widely applied in positioning systems in the automobile industry to measure angular position and rotational speed. There are usually two ways to fulfill the measurement task. One way is to use the back-bias method [21,22,23,24], in which a permanent magnet is used to produce a bias magnetic field nearby the specimen. When the ferromagnetic specimen, such as a gear wheel, moves under the magnet, the magnetic field around the magnet changes. This field variation can be sensed by a magnetic sensor and used to calculate the angular displacement and rotation speed. Another way to implement measurement angular position and displacement is to use the spatial distribution of the magnetic field produced by a magnet [25,26,27,28,29,30,31]. When the relative position between a magnet and a magnetic sensor changes, the magnetic field picked up by the magnetic sensor changes accordingly. This information could be used to indicate the change in angular position and displacement.

Recently, an alternating current magnetic flux measurement (AC-MFM) method for ferromagnetic materials was proposed [32]. The AC-MFM sensor has the advantages of low cost and compact structure compared to optical sensors. They also have a higher tolerance to harsh environments than capacitive sensors. Compared with ECSs, the AC-MFM sensor does not require a high operating frequency; hence, the signal conditioning circuit is simpler. The AC-MFM method is based on measuring the magnetic flux, which is related to the displacement in a magnetic circuit. The principle of the AC-MFM method is similar to the detection of the loss of the metallic cross-sectional area of wire ropes [33,34,35]. However, in this method, the eddy currents induced by the AC excitation affect the accuracy, linearity and measurement range. Thus, a very low frequency was suggested. In some applications, such as ellipticity and straightness measurements of steel pipes, low-frequency AC-MFM sensors cannot meet the requirements for high-speed inspection.

To develop a displacement sensor suitable for more applications, a permanent magnet magnetic flux measurement (PM-MFM) method is proposed in this study. The principle of our proposal is to measure the magnetic flux in a bridge-structured magnetic circuit composed of four permanent magnets. Since the measurement is based on the change in magnetic reluctance in a magnetic circuit, the proposed displacement sensor can only be applied to ferromagnetic samples. This is a limitation of the proposed sensor when compared with a capacitive sensor, optical sensor or eddy current sensor.

The remaining sections of this paper are organized as follows: in Section 2, the relationship between the displacement and the magnetic flux is derived through analyses of an equivalent magnetic circuit; in Section 3, the finite element model is established to validate the relationship; in Section 4, a PM-MFM sensor is designed and used to measure displacement experimentally; in Section 5, a comparison was made between the AC-MFM sensor and the PM-MFM sensor. The results of the verification experiments indicate that the output of our prototype sensor changes linearly with the reciprocal of the sum of a constant and displacement. The linear regression of the measured results shows that the correlation coefficients are larger than 0.999 across different measurement ranges. In addition, the sensor can be applied to various ferromagnetic materials without repetitive calibrations.

## 2. Sensing Principles

### 2.1. PM-MFM Method

The PM-MFM method is based on the relationship between the displacement and the magnetic flux. According to Hopkinson’s law [36,37], the magnetic flux Φ depends on the magnetic potential *F* and the magnetic reluctance *R*_m_:

$$\Phi =F/R_{m}$$

In the PM-MFM method, the magnetic potential difference comes from the permanent magnets. The magnetic reluctance includes the reluctance of the ferromagnetic materials and the air. In addition, the air reluctance in a magnetic circuit can be calculated as

$$R_{\mathrm{air}}=l/\mu_{0}S$$

where *l* is the length of the air gap (the displacement), *μ*_0_ is the permeability of air, and *S* is the cross-sectional area of the air gap. It can be observed that the magnetic flux changes with the variation in the air reluctance. Therefore, when a proper magnetic circuit is designed, the value of displacement can be obtained by measuring magnetic flux directly.

### 2.2. Bridge-Structured PM-MFM Sensor

**Figure 1:**
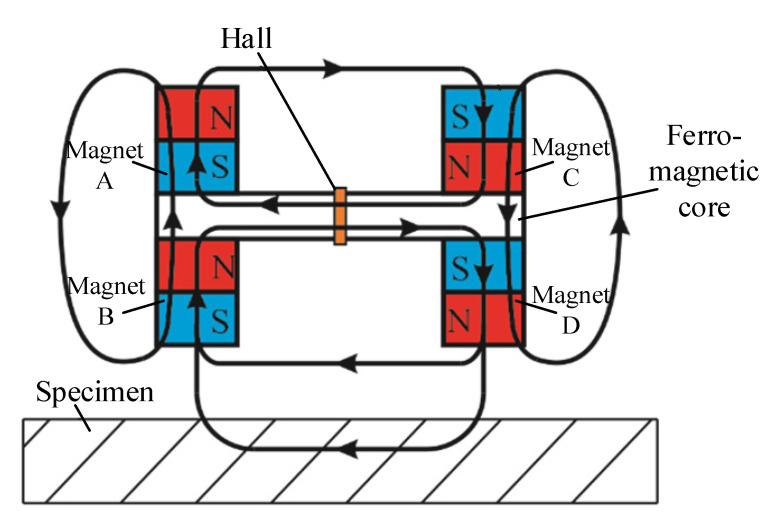
Schematic of the bridge-structured PM-MFM sensor.

A typical PM-MFM sensor is comprised of permanent magnets as excitation, ferromagnetic cores as connectors and magnetic sensors as reception. In this section, a bridge-structured PM-MFM sensor is introduced. As shown in Figure 1, the designed sensor consists of four permanent magnets and rectangular cores and a Hall sensor sandwiched between them.

**Figure 2:**
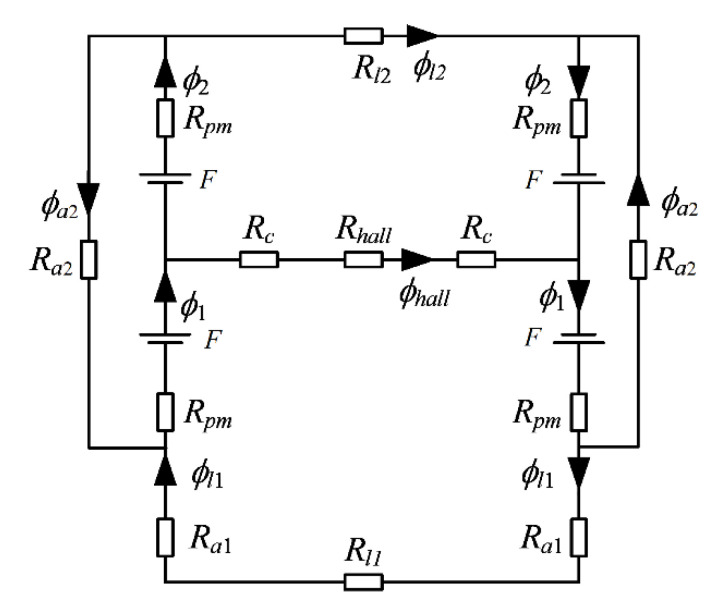
Equivalent magnetic circuit of the bridge-structured PM-MFM sensor.

To determine the relationship between the displacement and the magnetic flux through the Hall sensor, an equivalent magnetic circuit is established, with the leakage magnetic flux of each single permanent magnet neglected [38]. Figure 2 presents the simplified equivalent magnetic circuit with magnetic flux paths and magnetic reluctance identified.

According to the magnetic Kirchhoff’s Law and Hopkinson’s law, the following equations can be obtained:

(1) $$\left\{ \begin{array}{l} \Phi_{\mathrm{hall}}=\Phi_{1}-\Phi_{2}\\ \Phi_{a2}=\Phi_{1}-\Phi_{l1}=\Phi_{2}-\Phi_{l2}\\ \Phi_{1}R_{\mathrm{pm}}+\Phi_{2}R_{\mathrm{pm}}+\Phi_{a2}R_{a2}=2F\\ \Phi_{\mathrm{hall}}(R_{\mathrm{hall}}+2R_{c})+2\Phi_{1}R_{\mathrm{pm}}+\Phi_{l1}\left(2R_{a1}+R_{l1}\right)=2F\\ -\Phi_{\mathrm{hall}}(R_{\mathrm{hall}}+2R_{c})+2\Phi_{2}R_{\mathrm{pm}}+\Phi_{l2}R_{l2}=2F \end{array}\right.$$

where *F* is the magnetic potential of magnets, and $R_{\mathrm{pm}}$, $R_{c}$, $R_{a1}$, $R_{a2}$, $R_{l1}$ and $R_{l2}$ represent the magnetic reluctance of permanent magnets, ferromagnetic cores, the air gaps between sensor and specimen, the air between magnets A and B (or C and D), the specimen and the air between magnets A and C, respectively. $R_{\mathrm{hall}}$ represents the magnetic reluctance of the gap where the Hall element is placed. $\Phi_{\mathrm{hall}}$, $\Phi_{1}$, $\Phi_{2}$, $\Phi_{l1}$, $\Phi_{l2}$ and $\Phi_{a2}$ indicate the magnetic flux through the corresponding elements as shown in Figure 2.

Under the assumption that the reluctance of specimen $R_{l1}$ is much less than the air gap $R_{a1}$, the following can be derived from Equation (1):

(2) $$\Phi_{\mathrm{hall}}=\frac{2F}{C_{2}}\left(-R_{a2}+\frac{C_{2}R_{a2}R_{l2}+C_{1}R_{a2}}{C_{1}+2C_{2}R_{a1}}\right)$$

where

$$\begin{array}{ll} C_{1} & =2R_{\mathrm{pm}}^{2}R_{l}{}_{2}+4R_{\mathrm{pm}}^{2}R_{a}{}_{2}+2R_{\mathrm{pm}}\left(R_{\mathrm{hall}}+2R_{c}\right)R_{l}{}_{2}+4R_{\mathrm{pm}}\left(R_{\mathrm{hall}}+2R_{c}\right)R_{a}{}_{2}\\ & +2R_{\mathrm{pm}}R_{l}{}_{2}R_{a}{}_{2}+\left(R_{\mathrm{hall}}+2R_{c}\right)R_{l}{}_{2}R_{a}{}_{2} \end{array}$$

$$C_{2}=2R_{\mathrm{pm}}^{2}+2R_{\mathrm{pm}}R_{c}+2R_{\mathrm{pm}}R_{l}{}_{2}+2R_{\mathrm{pm}}R_{a}{}_{2}+\left(R_{\mathrm{hall}}+2R_{c}\right)R_{a}{}_{2}+R_{l}{}_{2}R_{a}{}_{2}$$

The reluctance of the air gap is $R_{a}{}_{1}=l/(\mu_{0}S_{\mathrm{arm}})$, where *l* is the displacement to be measured, $S_{\mathrm{arm}}$ is the cross-sectional area of the arm. Substitute it into Equation (2), the flux through the Hall sensor can be re-written as:

(3) $$\Phi_{\mathrm{hall}}=-\frac{2FR_{a2}R_{l2}}{C_{1}}\left(-1+\frac{2\mu_{0}S_{\mathrm{arm}}\left(C_{2}R_{l2}+C_{1}\right)}{C_{2}R_{l2}\mu_{0}S_{\mathrm{arm}}+2C_{1}R_{l2}l}\right)$$

Equation (3) can be simplified as:

(4) $$\Phi_{\mathrm{hall}}=C_{3}\left(-1+\frac{C_{4}}{l+C_{5}}\right)$$

by defining the following constants:

$$C_{3}=-2FR_{a2}R_{l}{}_{2}/C_{1}$$

$$C_{4}=\mu_{0}S_{\mathrm{arm}}\left(C_{2}R_{l2}+C_{1}\right)/C_{1}R_{l}{}_{2}$$

$$C_{5}=C_{2}R_{l}{}_{2}\mu_{0}S_{\mathrm{arm}}/2C_{1}R_{l}{}_{2}$$

According to the relation between the main magnetic flux and the air gap magnetic flux density [27], the magnetic flux density measured by the Hall element can be obtained

(5) $$B_{hall}=\frac{C_{3}}{k_{f}S}\left(-1+\frac{C_{4}}{l+C_{5}}\right)$$

where *k_f_* is the flux leakage factor, and *S* is the cross-sectional area of the ferromagnetic core. From Equation (5), we can notice that the magnetic flux density measured by Hall sensor changes linearly with the reciprocal of the sum of the displacement and the constant *C*_5_.

## 3. Simulation

In this section, a 3D finite-element model of the PM-MFM sensor is established to verify the linear relationship obtained in Equation (5). The sensor’s structure is the same as that presented in Section 2 and the parameters are given in Table 1. The length of the air gap between two rectangular cores is set to 1.5 mm. The simulation is developed in COMSOL. The magnetic flux density at the center of the sensor is recorded for different displacements in the range from 0.1 to 5 mm.

According to the relationship derived from Equation (5), the equation

(6) $$B=D_{1}+D_{2}\frac{1}{l+D_{3}}$$

is used to fit the data obtained from simulation, where *D*_1_, *D*_2_ and *D*_3_ are constants to be determined. In order to perform linear fitting, the fitting is performed to obtain the relationship between *B* and (*l* + *D*_3_)^−1^. Different values of *D*_3_ were chosen for the fitting of two different measurement ranges, namely, 0.1–5.0 mm and 0.6–4.7 mm, and the results are listed in Table 2. For the measurement range of 0.1–5.0 mm, *R*^2^ is higher than 0.999 when *D*_3_ ranges from 1.43 to 1.75 mm, indicating a good linearity. However, the maximum relative error is larger than 7.5%. To test the accuracy in a smaller measurement range, the fitting was also performed for displacements ranging from 0.6 to 4.7 mm. The *R*^2^ kept higher than 0.999, and the relative error is less than 2.83% when *D*_3_ was properly selected.

**Figure 3:**
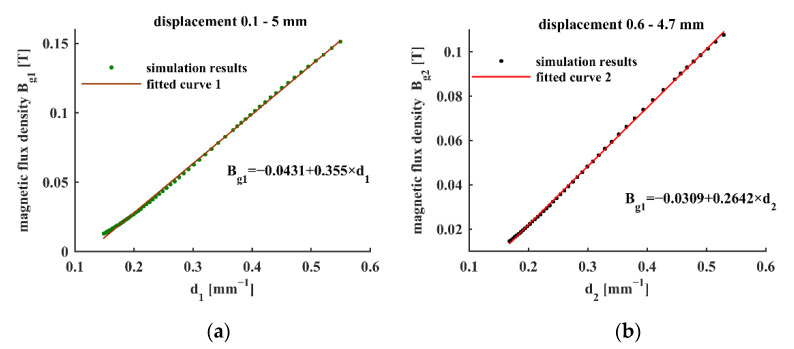
Linear fitting curves of magnetic flux density with *d*: *d*_1_ = (*l* + 1.72)^−1^ and *d*_2_ = (*l* + 1.27)^−1^ where *l* represents the displacement: (**a**) displacement range 0.1–5.0 mm (**b**) displacement range 0.6–4.7 mm.

The optimal fitting for the measurement range 0.1–5.0 mm and 0.6–4.7 mm is obtained when *D*_3_ equals 1.72 and 1.27, respectively. The results are shown mathematically in Equation (7) and graphically in Figure 3.

(7) $$\left\{ \begin{array}{l} B_{g1}=-0.0431+0.355\times \frac{1}{l\;+\;1.72},\;\mathrm{for}\;0.1<l<5.0\\ B_{g2}=-0.0315+0.269\times \frac{1}{l\;+\;1.27},\;\mathrm{for}\;0.6<l<4.7 \end{array}\right.$$

## 4. Experiments

### 4.1. Experimental Setup

**Figure 4:**
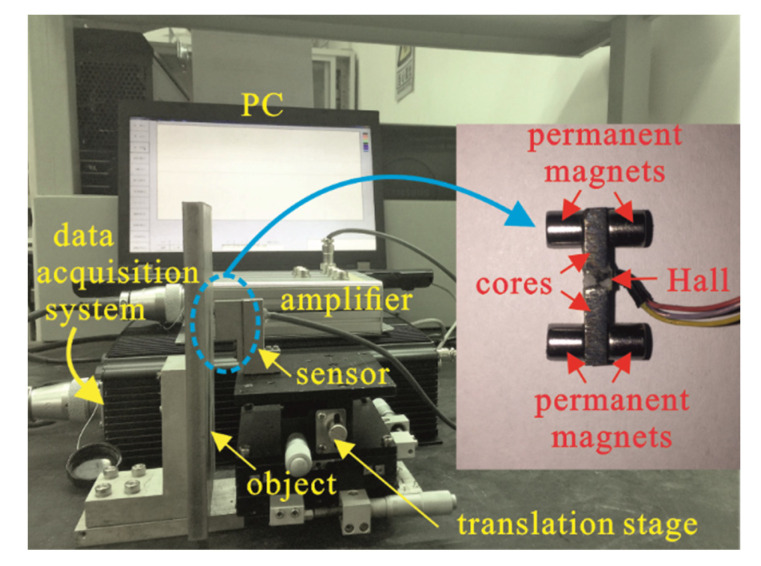
The experimental setup of the PM-MFM sensor; the inset shows the prototype PM-MFM sensor comprising four permanent magnets, two rectangular cores and a Hall sensor.

A prototype PM-MFM sensor composed of four permanent magnets, two rectangular cores and a Hall sensor, was manufactured as shown in Figure 4. The Hall sensor is placed at the air gap between two cores. The length of the air gap is 1.5 mm, which is the same as the thickness of the Hall sensor. The four permanent magnets adhered to the rectangular cores form a bridge-structured sensor. The packaged sensor is then fixed on a translation stage, with the measured object fixed in parallel. The measured object is a 250 mm (length) × 100 mm (width) × 50 mm (height) plate made of 45# steel. The gain of the amplifying circuit is set to 10. Parameters of the bridge-structured sensor are listed in Table 3.

### 4.2. Experimental Results

To verify the feasibility of the PM-MFM method, the displacement of the steel plate is measured in the range from 0.1 mm to 6.0 mm, with a step of 0.1 mm. The results are analyzed using linear regression. As listed in Table 4, the correlation coefficient *R*^2^ and the maximum relative error *ε* are employed to evaluate the linear regression for different values of the constant *D*_3_.

From Table 4, we can observe that the *R*^2^ values are larger than 0.99 and even 0.999 in some cases. For the displacement range 0.1–6.0 mm, the results show a good linearity while the maximum relative error is greater than 9.75%. When the displacement range is narrowed down to 0.5–5.0 mm, the results become better, showing a similar tendency as the simulation results. With *R*^2^ greater than 0.9995, and the maximum relative error decreases to 4.35% when C_3_ is 0.95 mm.

**Figure 5:**
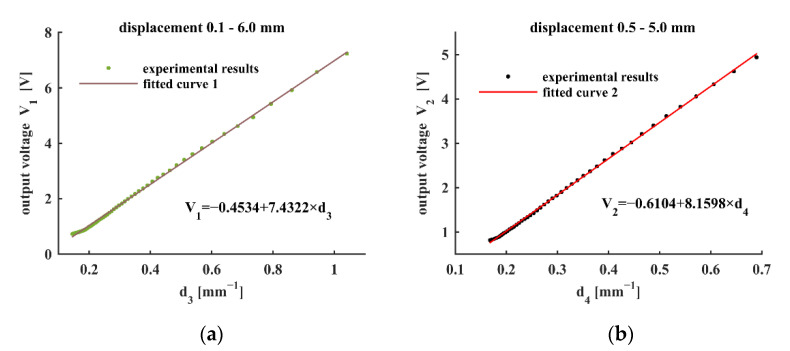
Linear fitting curves of magnetic flux density with *d*: *d*_3_ = (*l* + 0.86)^−1^ and *d*_4_ = (*l* + 0.95)^−1^ where *l* represents the displacement: (**a**) displacement range 0.1–6.0 mm; (**b**) displacement range 0.5–5.0 mm.

Figure 5 shows the linear fitting results for the experimental data. The lines in Figure 5a,b are the linear fitting curves for the displacement ranges 0.1–5.0 mm and 0.6–4.7 mm, respectively. The fitting results are mathematically expressed as follows:

(8) $$\left\{ \begin{array}{l} V_{1}=-0.4534+7.4322\times \frac{1}{l\;+\;0.86}\\ V_{2}=-0.6104+8.1598\times \frac{1}{l\;+\;0.95} \end{array}\right.$$

### 4.3. Influence of Specimen Property on the Measurement Result

For conventional displacement measurement sensors, such as ECSs, the sensor should be calibrated for each material because the electromagnetic properties have a significant influence on the measured results. However, for the PM-MFM method, the main factor influencing the measurement signal is the magnetic reluctance of the air gap. In a proper magnetic circuit, the magnetic reluctance of ferromagnetic materials can be neglected compared to air reluctance. Therefore, the variation in ferromagnetic materials will not considerably influence the result of the PM-MFM sensor. This conclusion can also be drawn from Equation (3); the output of the PM-MFM sensor mainly depends on the air gap between the sensor and the ferromagnetic object. This indicates that the PM-MFM sensor is compatible with different ferromagnetic materials. To prove this characteristic, five common ferromagnetic samples (45# steel, 20# steel, Q345 steel, 38Cr steel, and 40CrNi) were tested in the experiments. The displacement result for each sample was calculated by the previously obtained fitting equation *l* = 8.1598/(*V* + 0.6104) − 0.95, which is derived from Equation (8).

The displacement measurement results for different samples are listed in Table 5. As can be observed from Table 5, the fitting equation of 45# steel can fit the other four ferromagnetic materials. The measured displacements of each material have high accuracy with a maximum relative error of less than 5%. The results significantly show that the PM-MFM sensor is compatible with other ferromagnetic materials, and has great potential for use in industrial sectors.

### 4.4. Discussion

The results of the experiments showed that the PM-MFM method is effective for the displacement sensing. Moreover, the results present a very good linearity of the bridge-structured PM-MFM sensor. For the displacement range 0.5–5 mm, the output of the PM-MFM sensor changes linearly with the reciprocal of the sum of the displacement and a constant, with the correlation coefficient *R*^2^ larger than 0.9996. This characteristic makes it quite convenient for real-time data processing. On the other hand, the results of the testing on different samples verified that the change in the type of ferromagnetic sample did not affect the measured results much. The calibration for one ferromagnetic material can fit the other four. This feature implies that the sensor has no need of repetitive calibration when the measured object changes, and is useful for practical application in engineering and industry. However, the measured objects have to be ferromagnetic. 

The PM-MFM sensor has some limitations as well. First, the magnetic sensor in the PM-MFM sensor has to be placed in the air gap between the cores. This restricts the sensor’s size. In addition, the measurement range of the PM-MFM sensor is limited. In pursuit of a good linearity, the measurement range cannot be too small or too large. For small displacements, such as 0–0.5 mm, neglecting *R_l_*_1_ makes the calculated magnetic flux larger than the real one. For a large displacement, such as 5.0–6.0 mm, a part of the magnetic flux will go from magnet B to magnet D through the air between them and cause errors. Furthermore, the external magnetic source nearby the sensor could influence the measurement results. To reduce the influence of the environmental magnetic field, a ferromagnetic shell can be added to shield the external magnetic field.

## 5. Comparisons between PM-MFM Sensor and AC-MFM Sensor

Both the PM-MFM and AC-MFM sensors are based on the relation between the magnetic flux and the displacement. By designing a proper magnetic circuit, the relationship can be easily obtained. In the AC-MFM sensor, the magnetic field is excited by coils with a low-frequency alternating current, and the reception is achieved by using another coil. The receptive coil is wound around the core of the AC-MFM sensor to measure the magnetic flux. In the PM-MFM sensor, the magnetic field is supplied by the permanent magnets, and the magnetic sensor is a Hall element. The output of the AC-MFM sensor is the peak-to-peak value of the receptive coil, which is linear with the reciprocal of the displacement. In the PM-MFM sensor, the output is the voltage of a Hall sensor, which is linear with the reciprocal of the sum of the displacement and a constant. Both types have good linearity, but the PM-MFM sensor has a better one. 

In the PM-MFM sensor, the permanent magnets are used as excitation. As a result, the magnetic field in the PM-MFM sensor cannot be adjusted while it can be achieved by changing the excitation current in the AC-MFM sensor. However, with regard to the measurement range and the linearity, the PM-MFM sensor has advantages over the AC-MFM sensor. During the measurement of the AC-MFM sensor, the alternating current will introduce eddy currents in the measured objects, which impedes the change in the primary magnetic field and affects the linearity. Therefore, to diminish the effects of eddy currents, the operating frequency has to be very low. In the PM-MFM sensor, there are no eddy currents in the measured objects; hence, the PM-MFM sensor has better linearity and a larger measurement range. More importantly, the applications of the AC-MFM method are limited. In high-speed inspections such as the ellipticity measurement of coiled tubings, the measurement speed is required to reach 1 m/s. The operating frequency of the AC-MFM sensor in research [21] is 100 Hz, and the distance between the adjacent measured points is greater than 10 mm. As a result, the measured results are inaccurate. On the contrary, the PM-MFM sensor’s measurement is not restricted by the movement of the measured objects. In conclusion, both the AC-MFM sensor and the PM-MFM sensor have excellent performance, but the PM-MFM sensor has more advantages, especially in high-speed applications.

## 6. Conclusions

In this paper, a new displacement sensing method based on magnetic flux measurement was proposed. Different from the AC-MFM method, the PM-MFM method is propitious for high-speed measurements. A bridge-structured PM-MFM sensor was designed and tested. The results verified the feasibility of the PM-MFM method and the linear relation between the sensor’s output and the reciprocal of the sum of the displacement and a constant. A linear regression analysis of the measured results showed that the correlation coefficient is greater than 0.9995, in the measurement range of 0.5–5 mm. A comparison experiment showed that the PM-MFM sensor is compatible with different ferromagnetic materials, with a maximum relative error of less than 5%, demonstrating the potential of the PM-MFM sensor for use in industrial sectors.

## Figures and Tables

**Table 1:** Parameters of the model.

	Size (mm)	Material
Permanent magnet	2.5 (radius) × 3 (height)	NdFeB
Ferromagnetic core	6.75 (length) × 5 (width) × 2 (height)	45# steel
Steel plate	20 (length) × 10 (width) × 3 (height)	45# steel

**Table 2:** Linear regression of the simulation results.

0.1–5.0 mm			0.6–4.7 mm		
*D*_3_ (mm)	*R* ^2^	*ε* (%)	*D*_3_ (mm)	*R* ^2^	*ε* (%)
1.43	0.9990	38.83	1.04	0.9990	6.04
1.45	0.9991	36.60	1.09	0.9993	5.34
1.48	0.9992	34.37	1.14	0.9994	4.64
1.52	0.9993	28.78	1.19	0.9996	3.94
1.57	0.9993	23.18	1.24	0.9996	3.25
1.60	0.9993	19.82	1.27	0.9997	2.83
1.65	0.9992	14.21	1.34	0.9996	3.49
1.72	0.9991	7.50	1.39	0.9996	3.93
1.75	0.9990	7.82	1.50	0.9993	4.71
1.80	0.9988	8.32	1.60	0.9990	5.47

**Table 3:** Parameters of the PM-MFM sensor.

	Size (mm)	Material
Permanent magnet	5 (radius) × 5 (height)	NdFeB
Ferromagnetic core	10 (length) × 6 (width) × 3 (height)	45# steel

**Table 4:** Linear regression of the experimental results.

0.1–5.0 mm			0.6–4.7 mm		
*D*_3_ (mm)	*R* ^2^	*ε* (%)	*D*_3_ (mm)	*R* ^2^	*ε* (%)
0.76	0.9981	26.43	0.83	0.9989	7.11
0.78	0.9984	22.69	0.86	0.9991	6.34
0.80	0.9987	18.91	0.89	0.9993	5.57
0.82	0.9989	15.09	0.92	0.9995	4.80
0.84	0.9991	11.22	0.95	0.9996	4.35
0.86	0.9992	9.75	0.98	0.9996	4.77
0.88	0.9992	10.33	1.01	0.9996	5.17
0.90	0.9992	10.88	1.04	0.9996	5.55
0.92	0.9991	11.40	1.07	0.9993	5.91
0.94	0.9990	11.89	1.10	0.9990	6.24

**Table 5:** Displacement measurement for different materials.

Real Displacement	Measured Displacement					Average Value	Maximum Error (%)
	45#	20#	Q345	38Cr	40CrNi		
0.5	0.520	0.517	0.519	0.523	0.522	0.520	4.60
0.8	0.797	0.782	0.790	0.805	0.797	0.794	2.25
1.1	1.085	1.071	1.078	1.092	1.086	1.080	2.64
1.4	1.388	1.390	1.389	1.395	1.404	1.393	0.86
1.7	1.690	1.673	1.676	1.696	1.681	1.683	1.59
2.0	1.985	1.975	1.972	1.987	1.996	1.983	1.40
2.3	2.300	2.317	2.286	2.345	2.337	2.317	1.96
2.6	2.597	2.613	2.588	2.624	2.610	2.606	0.92
2.9	2.935	2.926	2.918	2.952	2.957	2.938	1.97
3.2	3.255	3.242	3.270	3.255	3.260	3.256	2.19
3.5	3.557	3.567	3.528	3.550	3.537	3.548	1.91
3.8	3.849	3.793	3.821	3.871	3.889	3.845	2.34
4.1	4.149	4.117	4.024	4.181	4.158	4.126	1.98
4.4	4.435	4.417	4.410	4.460	4.449	4.434	1.36
4.7	4.645	4.664	4.633	4.657	4.649	4.650	1.43
5.0	4.783	4.835	4.795	4.848	4.823	4.817	4.34

## Data Availability

Research data supporting this publication are available upon contacting authors by email.
